# The Collaborative Search by Tag-Based User Profile in Social Media

**DOI:** 10.1155/2014/608326

**Published:** 2014-06-11

**Authors:** Haoran Xie, Xiaodong Li, Jiantao Wang, Qing Li, Yi Cai

**Affiliations:** ^1^Department of Computer Science, City University of Hong Kong, Kowloon, Hong Kong; ^2^Department of Computer Science, Hong Kong Baptist University, Kowloon, Hong Kong; ^3^Multimedia Software Engineering Research Centre, City University of Hong Kong, Kowloon, Hong Kong; ^4^School of Software Engineering, South China University of Technology, Guangzhou 510006, China

## Abstract

Recently, we have witnessed the popularity and proliferation of social media applications (e.g., Delicious, Flickr, and YouTube) in the web 2.0 era. The rapid growth of user-generated data results in the problem of information overload to users. Facing such a tremendous volume of data, it is a big challenge to assist the users to find their desired data. To attack this critical problem, we propose the collaborative search approach in this paper. The core idea is that similar users may have common interests so as to help users to find their demanded data. Similar research has been conducted on the user log analysis in web search. However, the rapid growth and change of user-generated data in social media require us to discover a brand-new approach to address the unsolved issues (e.g., how to profile users, how to measure the similar users, and how to depict user-generated resources) rather than adopting existing method from web search. Therefore, we investigate various metrics to identify the similar users (user community). Moreover, we conduct the experiment on two real-life data sets by comparing the *Collaborative* method with the latest baselines. The empirical results show the effectiveness of the proposed approach and validate our observations.

## 1. Introduction

With the rapid development of web communities, we have witnessed the popularity and proliferation of social media applications in web 2.0 era, which allow the user to annotate and share various kinds of resources like web pages (Delicious (http://www.delicious.com/)), movies (Movielens (http://www.movielens.org/)), and images (Flickr (http://www.flickr.com/)). On one hand, the tremendous user-generated data provides the opportunity to easily communicate and share information with each other; on the other hand, such a big volume of data results in the problem of information overload to the users. Facing such a tremendous volume of data, it is a big challenge to assist the users to find their desired data.

To attack this critical problem, we propose the collaborative search approach in this paper. The core idea is that similar users may have common interests so as to help users to find their demanded data. Similar research [1–3] has been conducted on the user log analysis in web search. However, the rapid growth and change of user-generated data in social media require us to discover a brand-new approach to address the unsolved issues (e.g., how to profile users, how to measure the similar users, and how to depict user-generated resources) rather than adopting existing method from web search. Therefore, we investigate the following research questions in this paper:

- how to depict users and resources in the social media;
- how to measure the user similarity in the social media;
- how to assist users to find their interested data (resources) by similar users in the social media.

The remaining parts of this paper are structured as follows. In Section 2, the related works on collaborative search and social media are reviewed. We introduce the framework of the collaborative search for social media in Section 3. The experiments are conducted and the corresponding results are analyzed in Section 4. Finally, we summarize our work and discuss the potential directions for future research in Section 5.

## 2. Related Works

In this section, we review relevant works in the areas of collaborative search and social media.

### 2.1. Collaborative Search

Collaborative search (a.k.a., social search) has been intensively studied to facilitate the search performance in the web by incorporating similar user search behaviors. In [4], R. B. Almeida and V. A. F. Almeida devised a community-aware search engine, which incorporated community information as another evidence of relevance and improved the conventional content-based ranking strategies up to 48% of the average precision. Park and Ramamohanarao [1] proposed a popularity score for multiresolution community to generalize and improve PageRank algorithm for web search. Smyth [2] deployed a community-based search engine to collect search behavior for a community (e.g., a department) and found that the search quality can be significantly improved by the community members. Moreover, McNally et al. [3] described the results of real user study, which demonstrated the benefits of a collaborative search method (called HeyStaks) making use of personalization and social networking. In [5], HeyStaks was further enhanced and improved by the recommendation method and the reputation model in terms of the click-through rate. Morris et al. [6] explored the design space for collaborative search systems on interactive tabletops. Boydell and Smyth [7] described a technique for summarizing search results that harnesses the collaborative search behavior of communities of like-minded searchers to produce snippets that are more focused on the preferences of the searchers. Ju and Xu [8] proposed a novel collaborative recommendation approach based on users' clustering by using artificial bee colony algorithm. Xue et al. [9] developed a user language model for the personalized collaborative search, so that the behaviors of the group users can be utilized to improve the search performance. Fu et al. [10] exploited the characteristics of local communities to facilitate collaborative recommendations. Cai et al. [11] further improve the conventional collaborative filtering methods by borrowing the idea of “object typicality” in cognitive science.

### 2.2. Social Media

In this subsection, we mainly focus on one mainstream of social media applications: collaborative tagging systems. Previous research on the collaborative tagging system can be mainly divided into two classes. One is trying to find the main patterns and characteristics of the user-generated tags and resources in such social media communities. In [12], the tag generation and usage patterns were investigated and analyzed by Golder and Huberman. To reveal the power of the tags, Bischoff et al. [13] studied various aspects of the social tagging throughout a comprehensive survey on many real tagging data sets. Moreover, Gupta et al. [14] investigated and summarized the main patterns of tagging behaviors and the popular tagging techniques. Carmel et al. [15] presented a folksonomy-based term extraction method, called tag-boost, which boosts terms that are frequently used by the public to tag content. Wei et al. [16] analyzed and studied the cooperation rate in cooperation social networks via a two-phase Heterogeneous Public Goods Game (HPGG) model. Ye et al. [17] studied the feasibility of social network research technologies on process recommendation and built a social network system of processes based on the features' similarities. The other class is to apply these characteristics and patterns in various applications (e.g., social media resource search or recommendation). Bao et al. [18] presented two novel algorithms called SocialSimRank (SSR) and SocialPageRank (SPR) by incorporating social annotation to facilitate web search. Three approaches (naive, cooccurrence, and adaptive) were proposed by Michlmayr and Cayzer [19] to construct tag-based profiles and assist in information access. Xu et al. [20] measured the semantic relatedness between Flickr images from the tag-based perspectives. Balali et al. [21] presented a supervised approach to predicting and reorganizing the hierarchical structure of conversation threads for user-generated text in social media. The tag-based profiles were further studied and investigated to facilitate personalized search [22–24]. Furthermore, a source-initiated on-demand routing algorithm, which can assist users to communicate in mobile wireless sensor network, was proposed by Mao and Zhu [25].

## 3. Methodology

In this section, we will introduce and discuss the proposed collaborative search method for social media. First of all, the research problem is formulated so that the clear picture of the methodology is given. Then, the methodology can be further divided into three subprocesses, which are user and resource profiling, user community discovery, and collaborative ranking.

### 3.1. Problem Formulation

Intuitively, the collaborative search is to consider the search history of similar users as evidence of relevance and rerank the resources [3, 5]. Specifically, the research problem of collaborative search can be formulated as a mapping function *θ* as follows:

(1) $$\begin{array}{c} \theta :U\times Q\times C\times R⟶S, \end{array}$$

where *U* is the set of users, *Q* is the set of queries, *C* is the set of user communities (similar user clusters), and *R* is the set of resources; the ultimate goal of function *θ* is to map the above four elements to ranking score *S*. In the next three subsections, we will detail how to model user and resource, discover similar users, and perform the collaborative search.

### 3.2. User and Resource Profiling

To depict the user and resource, we adopt the bag-of-tags (BOT) paradigm to construct user and resource profiles, which is similar to our previous research in [23]. The paradigm is mainly based on the assumption that the tags used by user (or annotated to resource) reflect the user's interest (or resource feature) to some extent. Formally, the user and resource profiles are defined as follows.

**Definition 1 .**

The* user profile* of user *a* is a vector of tag : value pairs, which is denoted by ${\vec{U}}_{a}$ as follows:

(2) $$\begin{array}{c} {\vec{U}}_{a}=\left(t_{a,1}:v_{a,1},t_{a,2}:v_{a,2},\ldots ,t_{a,n}:v_{a,n}\right), \end{array}$$

where *t*_*a*,*x*_ is a tag used by user *a*, *n* is the total amount of tags used by this user, and *v*_*a*,*x*_ means the degree of interest for user *a* on this tag *t*_*a*,*x*_. Similarly, the resource profile is also defined by the BOT paradigm below.

**Definition 2 .**

The* resource profile* of resource *i* is also a vector of tag : value pairs, which is denoted by ${\vec{R}}_{i}$ as follows:

(3) $$\begin{array}{c} {\vec{R}}_{i}=\left(t_{i,1}:w_{i,1},t_{i,2}:w_{i,2},\ldots ,t_{i,m}:w_{i,m}\right), \end{array}$$

where *t*_*i*,*y*_ is a tag annotated to the resource *i*, *m* is the total number of tags annotated to this resource, and *w*_*i*,*y*_ indicates the degree of relevance for tag *t*_*i*,*y*_ to the resource. The weight of each in both user and resource profiles can be obtained by various methods (e.g., tag-frequency (TF) [26], tag-frequency and inverse resource frequency (TF-IRF) [22], best match 25 (BM 25) [27], and normalized tag-frequency (NTF) [23]). To compare and find the best paradigm for our problem, we compare these different paradigms in the experiment (see Section 4).

### 3.3. User Community Discovery

Users have similar interests and/or intentions usually form user groups (communities) explicitly and implicitly; the community-based information can be adopted and utilized to improve the efficiency and effectiveness of various user navigational behaviors [24, 28]. There are many existing techniques (e.g., the topic model [29], semantic space [30], and Gaussian mixture model [31]) that can be employed to discover user community. However, the main shortage for these community discovering approaches is that the time complexities of method are exponentially increased (e.g., Θ(*n* ^*k*^) in [29], where *n* is the number of tags and *k* is the number of communities), which are very time consuming [24] and unapplicable in current big data era. To tackle this problem, we propose a lightweight method to discover the user community with the acceptable level of time complexity. The core idea is to precluster off-line firstly and then discover the user community for the user according to his/her current issued query and user profile.

#### 3.3.1. Off-Line Clustering

The purpose of off-line stage is to precluster the similar users and classify them into some user communities. The existing clustering approaches [29–31] can be adopted in this step as it performs off-line. However, the performance of various clustering methods has been studied in [24]. Therefore, we employ a conventional clustering method K-means to clearly investigate the performance of various user similarity measurements. Intuitively, a straightforward method is to adopt Jaccard and Ochiai coefficient as follows:

(4) $$\begin{array}{c} {\text{Sim}}_{J}\left({\vec{U}}_{a},{\vec{U}}_{b}\right)=\frac{\left|{\vec{U}}_{a}\cap {\vec{U}}_{b}\right|}{\left|{\vec{U}}_{a}\cup {\vec{U}}_{b}\right|},\\ {\text{Sim}}_{O}\left({\vec{U}}_{a},{\vec{U}}_{b}\right)=\frac{\left|{\vec{U}}_{a}\cap {\vec{U}}_{b}\right|}{\sqrt{|{\vec{U}}_{a}|\times \left|{\vec{U}}_{b}\right|}}. \end{array}$$

For the purpose of clustering by K-means, the above similarities are required to be converted to distance (e.g., using Dist() = (1/Sim()) − 1). Since these measurements focus on the tag and neglect the relevance of each tag in the user profile, they are named as* tag-level distance*. If we focus on the degree of relevance, the Euclidean distance and Manhattan distance (named as* value-level distance*) can be used as follows:

(5) DistE(U→a,U→b)=∑i,j=(1,1)(na,nb)(va,i−vb,j)2,ppppppppppp(∀(i,j),ta,i=tb,j),DistM(U→a,U→b)=∑i,j=(1,1)(na,nb)|va,i−vb,j|,pp(∀(i,j),ta,i=tb,j).

In our earlier work [23], we have found that matching in both tag-level and value-level can contribute to finding relevant resources. Thus, we propose* hybrid-level distance* by integrating distances in tag-level and value-level as follows:

(6) $$\begin{array}{c} {\text{Dist}}_{H}\left({\vec{U}}_{a},{\vec{U}}_{b}\right)=e^{(1-{\text{Sim}}_{J}({\vec{U}}_{a},{\vec{U}}_{b}))/{\text{Sim}}_{J}({\vec{U}}_{a},{\vec{U}}_{b})+{\text{Dist}}_{E}({\vec{U}}_{a},{\vec{U}}_{b})}, \end{array}$$

where $(1-{\text{Sim}}_{J}({\vec{U}}_{a},{\vec{U}}_{b}))/{\text{Sim}}_{J}\left({\vec{U}}_{a},{\vec{U}}_{b}\right)$ and ${\text{Dist}}_{E}({\vec{U}}_{a},{\vec{U}}_{b})$ are the distances in tag-level and value-level, respectively (there are other combinations for the hybrid-level distance and we select one of them to illustrate the usefulness of the hybrid of both tag-level and value-level distances). After selecting a particular distance (similarity) measurement above, K-means is then performed to discover *k* user communities (clusters). Formally, the community profile is depicted by members and their relevance as follows.

**Definition 3 .**

The* community profile* of community *k* is a vector of member : value pairs, which is denoted by ${\vec{C}}_{k}$ as follows:

(7) $$\begin{array}{c} {\vec{C}}_{k}=\left({\vec{U}}_{k,1}:d_{k,1},{\vec{U}}_{k,2}:d_{k,2},\ldots ,{\vec{U}}_{k,p}:d_{k,p}\right), \end{array}$$

where ${\vec{U}}_{k,z}$ is the user profile of community member (user) *z* and *d*_*k*,*z*_ is the distance of the user to the centroid of community *k*.

#### 3.3.2. On-Line Discovering

In off-line clustering stage, a user is classified to a particular user community according to his/her user profile. While in on-line discovering stage, we cannot fix the user to his/her preallocated community as the search context may be different or even totally irrelevant to it [32]. To avoid this case, we firstly compare the query with the user profile to examine whether the current query context is relevant to the user profile or not. Then, we discover a new user community for the user if the current issued query is not relevant to his/her current community. Finally, the user profile is updated by the query terms (tags) accordingly. The detailed algorithm is shown in Algorithm 1. Note that the time complexity of the algorithm is quite acceptable as it only has the time complexity Θ(*k*) and is much faster and more scalable than the on-line methods of Θ(*n* ^*k*^).

### 3.4. Collaborative Ranking

The last stage is to obtain the ranking score for the resource. Since the user (*U*), query (*Q*), community (*C*), and resource (*R*) are obtained and defined, we can adopt cosine measurement as the ranking function *θ* as

(8) θ(U→i,q→,C→o,R→x)  =R→x·U→i||R→x||||U→i||·R→x·q→||R→x||||q→||·R→x·U→o∗||R→x||||U→o∗||,

where ${\vec{U}}_{i}$ and ${\vec{R}}_{x}$ are given in Definitions 1 and 2, ${\vec{C}}_{o}$ and ${\vec{U}}_{o}^{*}$ are obtained in Algorithm 1, and $\vec{q}$ is the query. The greater value of *θ* function for a resource indicates the higher relevance to user interests and his/her current search intentions.

## 4. Experiment

In this section, we conduct the experiment on FMRS [32] and Movielens (http://www.grouplens.org/node/73) data sets to evaluate the performance of the proposed method.

### 4.1. Data Sets

The details of the two data sets are shown in Table 1. The main reason for selecting these two data sets is that they are in different domains (cooking recipes and movies) and they have different scales (10^4^ versus 10^7^ tags) so that we can examine the performance of the proposed method in both large and small scales for multiple domain applications in social media. To evaluate the proposed method, we split the data sets into 80% and 20% as training and testing sets, respectively. In training stage, the profiles and models are learned, while we examine whether the learned models can predict the right target resource by the given query terms (tags) from the testing set in the testing stage.

### 4.2. Metrics

Two widely adopted metrics are used in the experiments, which are *P*@*N* (Precision @*N*) [33] and MRR (Mean Reciprocal Rank). *P*@*N* is mainly to measure the accuracy of a particular search strategy, which is given as follows:

(9) $$\begin{array}{c} P@N=\overset{n}{\underset{i=1}{\sum }}\frac{p\left(q_{i}\right)}{n},\\ p\left(q_{i}\right)=\{\begin{array}{cc} 1, & \text{if}\;\;p\left(R_{i}^{q}\right)\leq N\\ 0, & \text{if}\;\;p\left(R_{i}^{q}\right)>N, \end{array} \end{array}$$

where *p*(*R*_*i*_^*q*^) is the position of target resource for query *q*_*i*_ and *n* is the total number of tuples in the testing set. The metric MRR reflects how quickly a search strategy can assist users in finding their desired resources, which is given as follows:

(10) $$\begin{array}{c} \text{MRR}=\frac{1}{n}\cdot \overset{n}{\underset{i=1}{\sum }}\frac{1}{\mathrm{rank}\left(r_{i}^{q}\right)}. \end{array}$$

### 4.3. Baselines

To verify the effectiveness of the proposed method, there are three state-of-the-art baselines for comparison. We denote the proposed method as “*Collaborative*” to simplify the notations. The abbreviations and details of the three baselines are introduced as follows.

*Profile-Based.* The profile-based personalized method was proposed in [27], which neglects the community-based information and only considers the relationships among the user profile, the resource profile, and the query.

*Community-Aware.* The community-aware resource search method [24] takes not only the user community but also the user and resource profiles into consideration. The main shortage of this approach is that the community discovering stage was performed on-line so that it is quite time consuming as discussed in Section 3.3.

*Social.* The social search method was proposed in [5] using similar user queries and the users' clicked resources as the evidence of relevance. The main difference between the social method and the collaborative search is that they are focusing on the tag-level, while the latter one takes both tag-level and value-level into consideration.

### 4.4. Overall Performance

**Figure 1:**
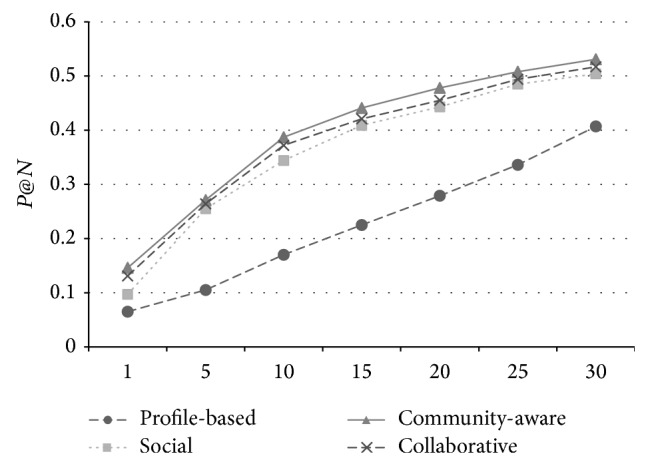
The performance of *P*@*N* on FMRS data set.

**Figure 2:**
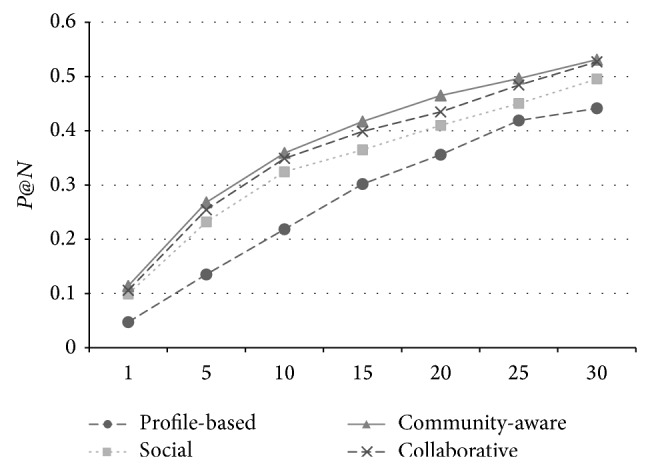
The performance of *P*@*N* on Movielens data set.

The overall performance of the metric *P*@*N* in FMRS and Movielens data sets is shown in Figures 1 and 2. We can find that the community*-*aware baseline achieves the best performance among all four methods with all *N* values (from 1 to 30), while the* Collaborative* method performs the second best (less accuracy from 0.7% to 2.3% with community-aware). This is mainly because the community-aware adopts the on-line clustering method which timely updated the user communities and the most relevant one can be obtained by the user. Note that the cost of on-line clustering is quite expensive (Θ(*n* ^*k*^)). Meanwhile, the off-line clustering of* Collaborative* only has the complexity with Θ(*k*). Moreover, it is observed that the social baseline, which only replies the tag-level profiles, is less accurate than both the* Collaborative* and community-aware ones. Therefore, we argue that considering both tag-level and value-level of the resource and user profiles will improve the search quality. Last but not least, the profile-based, which neglects the user community, has the worst achievement among all methods. It implies that the community-based information is quite useful to assist to find their relevant resources. Furthermore, we observe that the metric MRR has a similar trend to *P*@*N*, as shown in Table 2.

### 4.5. Alternative Paradigms and Distances

As we discussed in Sections 3.1 and 3.2, there are some existing alternative paradigms for user and resource profiling and other distance measurements (tag-level, value-level, and hybrid-level) for user similarity measurement. To investigate the impact of these alternative techniques, we compare their MRR values with various settings in* Collaborative* method. As shown in Table 3, the paradigm of NTF (the values with bold) has the best MRR performance. This result is consistent with our previous study in paradigm comparison [23]. Furthermore, we investigate the various distance measurements for user similarity. According to Table 4, we can observe that the hybrid distance is the most suitable one (with the bold values). It verifies our observation that the hybrid distance is a good tradeoff between tag-level and value-level distances. The value-level distance (Euclidean and Manhattan) gains the second best performance, which indicates that value-level distances are more precise than tag-level ones. We can further observe that the MRR value in tag-level distance (Jaccard and Ochiai) has a similar performance with social baseline, which also focuses on tag-level only.

## 5. Conclusion

In this research, we have proposed a lightweight user clustering method to find similar users in social media. The performance of collaborative search based on this clustering method is a bit less accurate than the on-line clustering community approach. However, the trade-off here is that we have gained much more scalability with less time complexity (from Θ(*n* ^*k*^) to Θ(*k*)). Moreover, the various distance measurements in three levels (tag-level, value-level, and hybrid-level) have been investigated. We believe that the proposed hybrid distance metric is the most suitable to measure the user similarity. Furthermore, we have confirmed the performance with NTF paradigm, which is the proper one to construct user and resource profiles. In the future study, we plan to find out the most important feature of score function *θ* so as to further improve the search quality in social media.

## Figures and Tables

**Algorithm 1:**
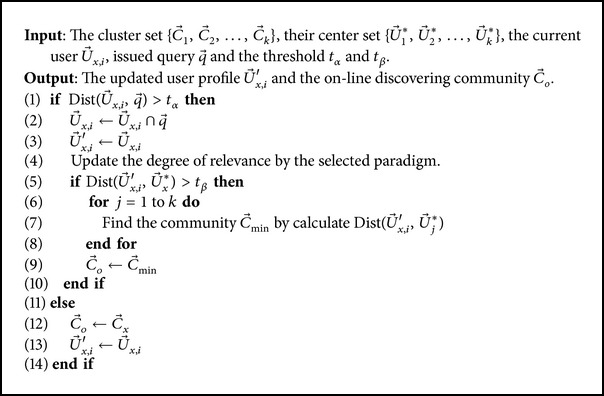
Algorithm of on-line community discovery.

**Table 1:** The details of FMRS and Movielens data set.

	Users number	Resources number	Tags number	Domain
FMRS	203	500	7889	Cooking recipes
Movielens	71567	10681	10000054	Movies

**Table 2:** The performance of MRR on FMRS and Movielens data sets.

	Profile-based	Social	Community-aware	Collaborative
FMRS	0.183	0.221	0.240	*0.229 *
Movielens	0.109	0.178	0.213	*0.194 *

**Table 3:** The performance with different paradigms in *Collaborative* on two data sets.

	TF	TF-IRF	BM 25	NTF
FMRS	0.188	*0.215 *	0.196	0.229
Movielens	0.167	*0.189 *	0.173	0.194

**Table 4:** The performance with different metrics in *Collaborative* on two data sets.

	Tag-level		Value-level		Hybrid-level
	Jaccard	Ochiai	Euclidean	Manhattan	Hybrid
FMRS	0.218	0.220	*0.226 *	0.224	0.229
Movielens	0.175	0.179	0.186	*0.191 *	0.194
